# Hidden in Plain Sight: Unmasking a Pseudoaneurysm Without Contrast

**DOI:** 10.3390/diagnostics16111652

**Published:** 2026-05-27

**Authors:** Martina Visonà, Giorgio Del Re, Raffaella Motta, Roberto Stramare, Chiara Giraudo

**Affiliations:** Unit of Advanced Clinical and Translational Imaging, Department of Cardiac, Thoracic, Vascular Sciences and Public Health—DCTV, University of Padova, 35122 Padova, Italy; martina.visona@studenti.unipd.it (M.V.);

**Keywords:** pseudoaneurysm, angiography computed tomography, Magnetic Resonance Imaging, contrast medium

## Abstract

Pseudoaneurysms are the outpouching of arterial walls confined to one or two layers, unlike true aneurysms which affect all three arterial wall layers (the intima, media, and adventitia). If peripheral, like in the femoral artery, ultrasound can be used for its diagnosis, but particularly in cases of large pseudoaneurysms of the aorta, an accurate characterization with CT angiography is recommended for treatment planning. In patients who cannot undergo an injection of contrast medium, as with our patient who was affected by severe renal insufficiency and had incidental evidence of an inhomogeneous lesion nearby the descending thoracic aorta at CT, the use of MR represents a valid alternative to diagnose the pseudoaneurysm. Indeed, by applying proper protocol using T2w True Fast Imaging with Steady-State Free Precession (TRUFI) and T1 Dixon sequences, we have been able to accurately characterize the pseudoaneurysm, ruling out other vascular findings such as intramural hematoma, or mediastinal lesions, such as undifferentiated pleomorphic sarcoma. This case highlights the value of MR without contrast for vascular assessment.

**Figure 1:**
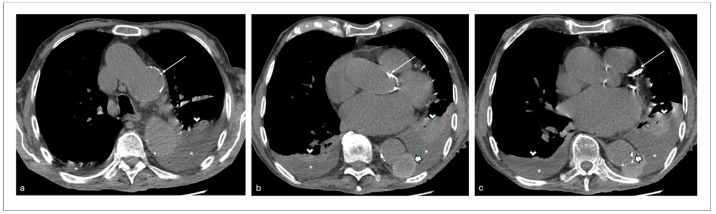
An 85-year-old man with severe renal insufficiency (serum creatinine level 266 µmol/L, normal range 59–104 µmol/L, and glomerular filtration rate 18 mL/min/1.73 m^2^) and without any history of recent trauma underwent a plain chest CT scan (CT; CT scanner General Electrics, GE, Revolution EVO 64, GE Healthcare, Chicago, IL, USA) for persistent cough and shortness of breath for months. The axial unenhanced chest CT revealed bilateral pleural effusion (white asterisks in (**a**–**c**)) and atelectasis (white arrowhead in (**a**–**c**)). Moreover, a large aneurysm of the aortic arch and diffuse vascular calcifications (thin white arrow in (**a**–**c**)) were evident. As an incidental finding, a round inhomogeneous lesion was detected near to the descending thoracic aorta (white arrow in (**b**)), with outpouching calcifications (white arrow in (**c**))). Given the well-defined, rounded shape, its location near the descending thoracic aorta, the displaced calcification, and the hyperdense signal, it was considered as a possible pseudoaneurysm. Although the hyperdense peripheral component, compatible with a hematoma, the smooth margins, the proximity to the aorta, and the outpouching calcifications were suggestive of a pseudoaneurysm, further assessment was needed. It should also be highlighted that the patient was recently checked with an echocardiography which assessed the presence of the aneurysm in the ascending aorta but did not mention the pseudoaneurysm. Since the patient could not undergo a contrast-enhanced examination to better characterize these findings, a thoracic MR was required.

**Figure 2:**
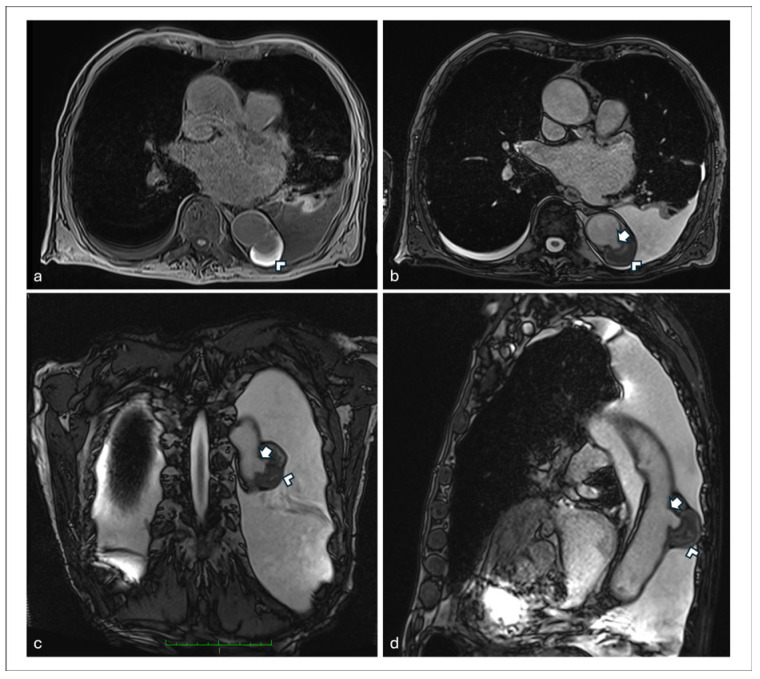
The patient was examined by a 1.5 T scanner (Siemens Magnetom Sola, Siemens Healthcare GmbH, Erlangen, Germany) and the protocol included axial and coronal T1w Dixon (3 mm slice thickness, TR 6.7 ms, TE 2.39 ms and TR 6.88 ms, TE 2.39 ms) and coronal, axial, and sagittal T2w True Fast Imaging with Steady-State Free Precession (TRUFI; 3 mm slice thickness, TR 673.53 ms, TE 2.3 ms). The MR clearly revealed the hyperintensity of the peripheral component, which was indicative of a hematoma (white arrowhead in (**a**)) on the T1w Dixon sequences, which then appeared hypointense in the axial, coronal, and sagittal T2 TRUFI images (white arrowhead in (**b**–**d**)); moreover, the TRUFI clearly showed the neck of the pseudoaneurysm (white arrow in (**b**–**d**)), indicating the direct communication between the lumen of the descending aorta and the lesion, allowing the diagnosis of a pseudoaneurysm. A hematoma within an aneurysm could have been considered as a differential diagnosis. Nevertheless, several features suggested that it was instead a pseudoaneurysm. Indeed, it had a disrupted calcification on the CT, a saccular outpouching rather than a fusiform shape, and it showed a narrow rather than a wide neck [1,2,3,4]. Moreover, in partially thrombosed aortic aneurysm the mural thrombus usually lines the wall, often appearing as peripheral concentric layers, while in pseudoaneurysms this often presents as a circulating sac or hematoma [1,2,3,4].

**Figure 3:**
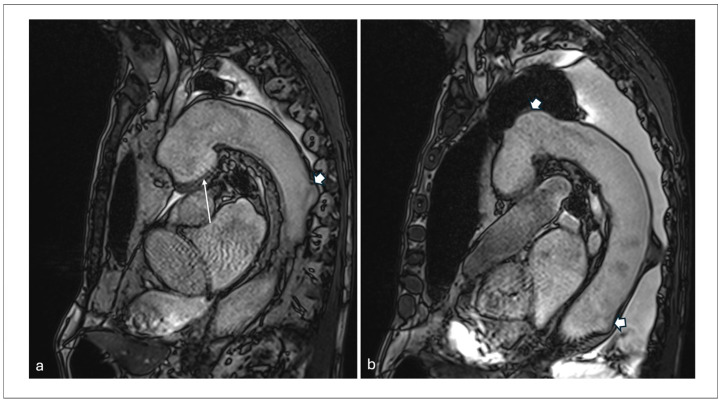
Sagittal TRUFI sequence demonstrating the multiple aneurysms along the thoracic aorta (white arrows in (**a**,**b**)), with the largest in the arch which was already seen in the CT (thin white arrow in (**a**)). Pseudoaneurysms or false aneurysms are vascular anomalies characterized by a disruption in one or two arterial wall layers, where the resulting hematoma is contained not by the vessel wall itself, but by the surrounding mediastinal soft tissues or a fibrous capsule formed from the clotting cascade [4,5,6]. Unlike true aneurysms, which involve all three layers of the aortic wall (intima, media, and adventitia), pseudoaneurysms are effectively “false” aneurysms that communicate with the aortic lumen through a neck or tear as in our case [4,5,6]. The main causes of pseudoaneurysms include blunt or penetrating trauma—often resulting from deceleration injuries at the aortic isthmus—infections such as mycotic aneurysms or tuberculosis, and iatrogenic injury at anastomotic sites following cardiac surgery [4]. Spontaneous dissection, fibromuscular dysplasia, and vasculitides should also be considered as etiology [4,5,6]. In our case, the patient did not report any thoracic trauma, and the presence of multiple aneurysms allows us to hypothesize fibromuscular dysplasia as etiology. While rare, the epidemiology of pseudoaneurysm is significant due to its high mortality. In fact, the risk of rupture is higher than that of a true aneurysm due to the lower support of the aneurysm wall. Diagnostic imaging is critical for diagnostic and therapeutic management [4,5,6,7]. If peripheral, like in the femoral artery, US can be useful for the diagnosis. Transthoracic echocardiography is severely limited by the acoustic window [2,8,9,10]. The descending thoracic aorta is located posteriorly, making it difficult to visualize clearly through lung tissue and ribs, which could explain why it was not mentioned in the echocardiography performed in our patient shortly before the CT. Even transesophageal echocardiography, while better, is invasive and has “blind spots” in the distal ascending aorta and proximal arch [2,8,9,10]. Therefore, especially in cases of large pseudoaneurysms of the aorta, an accurate characterization with CT angiography is recommended for treatment planning since it carries the advantages of rapid acquisition and high spatial resolution [1,2,3,4]; the appearance of pseudoaneurysms on unenhanced CT can be heterogeneous and not sufficient for proper assessment [4,5,6]. MR angiography, with contrast injection, is a valuable alternative without radiation exposure, allowing the distinction between slow vascular flow/stasis and an organized mural thrombus. In fact, in the early post-contrast phase, the patent lumen enhances immediately, while a thrombus remains hypointense. If the pseudoaneurysm is suspected to be “mycotic”, contrast-enhanced imaging can reveal perivascular enhancement and edema in the surrounding soft tissues, which indicates infection or inflammation [1,2,3,7,11]. MRI also serves in patients with renal impairment or contrast allergies, taking advantage of specific sequences like the abovementioned TRUFI [12,13,14]. In fact, TRUFI sequences play a valuable role in vascular assessment because they provide high-resolution, high-contrast images of blood vessels without requiring contrast medium injection [12,13,14]. Their steady-state free-precession technique produces bright-blood imaging that clearly delineates vessel lumen, flow patterns, and surrounding anatomy [12,14]. TRUFI sequences also offer rapid acquisition times and reduced motion sensitivity, allowing reliable visualization of major arteries and veins in dynamic regions such as the chest and abdomen [12,14]. After the MR, our patient was referred to the vascular surgeons of our tertiary center, who proposed a mini-invasive surgical procedure; however, the patient refused it. Currently, the patient is taking multiple medications including metoprolol, furosemide, allopurinol, darbepoetin alfa, and ketoanalogues. In conclusion, MRI allows for the accurate characterization of pseudoaneurysms, especially when contrast medium cannot be used.

## Data Availability

The original contributions presented in this study are included in the article material. Further inquiries can be directed to the corresponding author(s).

## Abbreviations

The following abbreviations are used in this manuscript:

MRI	Magnetic Resonance Imaging
CT	Computed Tomography
TR	Repetition Time
TE	Echo Time
TRUFI	True Fast Imaging with Steady-State Free Precession
